# Protective Effects of Diallyl Sulfide and Curcumin Separately against Thallium-Induced Toxicity in Rats

**DOI:** 10.22074/cellj.2016.3752

**Published:** 2015-07-11

**Authors:** Mohamed M. Abdel-Daim, Rania H. Abdou

**Affiliations:** 1 Department of Pharmacology, Faculty of Veterinary Medicine, Suez Canal University, Ismailia, Egypt; 2 Department of Forensic Medicine and Toxicology, Faculty of Veterinary Medicine, Suez Canal University, Ismailia, Egypt

**Keywords:** Garlic, Turmeric, Thallium Acetate, Oxidative Stress, Antioxidant

## Abstract

Thallium acetate (TI) is a cumulative poison intimately accompanied by an increase in reactive oxygen species (ROS) formation that represents an important risk factor for tissue injury and malfunction. This study aims to determine the possible hepatoprotective and antioxidant effects of diallyl sulfide (DAS) from garlic and curcumin from turmeric against TI-induced liver injury and oxidative stress (OS) in rats. This *in vivo* animal study divided rats into six groups of 8 rats per group. The first group received saline and served as the control group. The second and third groups received DAS or curcumin only at a dose of 200 mg/kg. The fourth group received TI at a dose of 6.4 mg/kg for 5 consecutive days. The fifth and sixth groups received DAS or curcumin orally 1 hour before TI intoxication at the same dose as the second and third groups. Liver integrity serum enzymes aspartate aminotransferase (AST), alanine aminotransferase (ALT), alkaline phosphatase (ALP), lactate dehydrogenase (LDH), and γ-glutamyltransferase (γ-GT) were evaluated. Serum and liver tissue homogenate lipid peroxidation and OS biomarkers were measured. The data were analyzed by one-way ANOVA followed by Duncan’s multiple range test for post hoc analysis using SPSS version 16. TI induced marked oxidative liver damage as shown by significantly (P≤0.05) elevated serum AST, ALT, ALP, LDH and γ-GT levels. There were significant (P≤0.05) increases in serum and hepatic malondialdehyde (MDA) and serum nitric oxide (NO) as well as decreased hepatic glutathione (GSH) and catalase (CAT) activities. There were significantly (P≤0.05) less serum and hepatic superoxide dismutase (SOD) and total antioxidant capacity (TAC). Pre-treatment with DAS or curcumin ameliorated the changes in most studied biochemical parameters. DAS and curcumin effectively reduced TI-induced liver toxicity.

## Introduction

Thallium acetate (TI) is a rare but widely discrete element. It is a cumulative poison broadly used by the semiconductor industry, in optical lenses, and for cardiac perfusion imaging. Furthermore, it is used in jewels, cement, photographic and electronic industries as well as its application as a rodenticide and as a medicine to treat ringworm, gonorrhea, syphilis and tuberculosis. In addition, TI acts as a potential constituent of growth media used to culture microorganisms (1, 2).

Acute TI poisoning usually occurs as a result of accidental, criminal or suicidal ingestion of this metal (3). TI toxicity is intimately accompanied by an increase in reactive oxygen species (ROS) formation, which represents an important risk factor for tissue injury and dysfunction (4). The liver is a vital organ which performs many essential metabolic functions and maintains body homeostasis (5). Excessive free radicals are generated during metabolism and may cause liver damage. In healthy animals there is an antioxidant defense system against ROS-mediated cell damage represented by endogenous antioxidants such as glutathione (GSH) and enzymatic scavengers such as superoxide dismutase (SOD) and catalase (CAT). These antioxidants neutralize, metabolize, or remove free radicals and protect the cells against oxidative damage (6-8).

Although synthetic antioxidants are widely used they have a number of adverse effects, whereas numerous medicinal plants have natural antioxidant activities. Their consumption is associated with low risk of certain diseases such as cancer with no health risk to consumers. This beneficial effect is attributed to their bioactive compounds as diallyl sulfide (DAS) exists in garlic and curcumin is present in turmeric, both of which have the ability to prevent cancer by acting as efficient free radical scavengers (9).

DAS is an active organic sulfur component derived from garlic, *Allium sativum*, that can suppress cytotoxicity induced by chemicals in animal models (10) and is well-known for its antioxidant properties (11-13). DAS and related compounds have been shown to suppress oxidative tissue damage by increasing SOD and GSH activities and decreasing malondialdehyde (MDA) levels in lung and kidney tissues (14-17).

Curcumin (diferuloylmethane), a yellow component of the spice turmeric obtained from the rhizome of Curcuma longa Linn is a persistent herb spread mainly throughout tropical and subtropical regions. Curcumin has strong antioxidant, antiinflammatory, anti-mutagenic and anti-carcinogenic properties (18-20). Curcumin can suppress lipid peroxidation and recover chemically-induced oxidative stress (OS) (21, 22) as well as increase xenobiotic detoxifying enzymes’ activities in both the liver and kidneys (23).

Numerous reports illuminate the effects of TI toxicity in relation to the formation of ROS (24- 26). However, no reports are available about the role of DAS and curcumin against TI-induced oxidative liver damage. The present study examines the possible protective and antioxidant effects of DAS and curcumin against TI-induced liver injury and OS in rats.

## Materials and Methods

### Chemicals

TI and DAS were purchased from Sigma Chemical Co. (St. Louis, Mo, USA). Curcumin was purchased from Bio Basic Inc. (Toronto, Canada). All kits used in the current study were purchased from Biodiagnostics Co. (Cairo, Egypt). All other chemicals were of analytical grade.

### Experimental animals

A total of 48 male adult albino rats (150-170 g) were obtained from the animal house of the National Central Institute, Dokki, Cairo, Egypt and allowed to acclimatize to their environment for one week prior to the start of the experiment. The rats were housed in stainless-steel cages (8 animals per cage) in the well ventilated animal house of the Department of Pharmacology, Faculty of Veterinary Medicine, Suez Canal University on a 12-hour light-dark cycle. Rats were fed on standard pellet and allowed free access to water.

### Experimental design

The current study experimental design and animal handling were approved by the Research Ethical Committee of the Faculty of Veterinary Medicine, Suez Canal University, Ismailia, Egypt (approval no. 20144). All precautions were made to minimize the rats’ suffering.

Rats were divided into six groups (n=8 per group). The first group was the control group that received corn oil orally and a saline i.p. injection. Groups 2 and 3 received DAS or curcumin only at 200 mg/kg body weight or 5 days. Group 4 received an i.p. injection of aqueous solution of TI for 5 days at 6.4 mg/kg body weight (BW, 1/5 of the rat’s lethal dose 50% (LD50), which is 32 mg/kg) (27). Groups 5 and 6 received oral DAS or curcumin dissolved in corn oil at 200 mg/kg BW, 1 hour before TI administration for 5 days. The doses of DAS and curcumin were chosen according to previous literatures (10, 28).

Blood was collected from postorbital plexus at 24 hours after the last TI dose, allowed to clot at room temperature and the serum was separated by centrifugation at 3000 rpm for 10 minutes, then used for biochemical estimation of liver integrity, lipid peroxidation and OS biomarkers. Rats then were sacrificed by ether anesthesia; the liver was removed from each rat and washed several times with normal saline before its use for biochemical estimations of lipid peroxidation and antioxidant biomarkers.

### Serum biochemical assay

The appropriate kits were used for the determination of serum alanine aminotransferase (ALT) and aspartate aminotransferase (AST) according to Reitman and Frankel (29) and alkaline phosphatase (ALP) according to Tietz et al. (30). These enzyme activities were defined as units/liter calculated directly from the absorbance values. Serum total protein was measured according to Lowry et al. (31). Lactate dehydrogenase (LDH) was determined or quantitated in serum according to the method of Babson and Babson (32). Gama glutamyltransferase (γ-GT) was evaluated according to Szasz (33). Cholesterol was measured according to Richmond (34) and Allain et al. (35). Total bilirubin was determined according to Schmidt and Eisenburg (36). Lipid peroxidation was evaluated by estimation of MDA according to Mihara and Uchiyama (37). Serum nitric oxide (NO) was determined according to Green et al. (38), while OS markers were assessed by estimation of SOD according to Nishikimi et al. (39) and total antioxidant capacity (TAC) according to Koracevic et al. (40).

### Liver lipid peroxidation and antioxidant status

The liver was homogenized (10% w/v) in ice-cold 0.1 M sodium-potassium phosphate buffer (pH=7.4). The homogenate was centrifuged at 3000 rpm for 15 minutes at 4˚C and the resultant supernatant used for assessments of different lipid peroxidation and OS markers. Liver tissue MDA was determined according to Mihara and Uchiyama (37), SOD according to Nishikimi et al. (39), CAT according to Aebi (41), reduced GSH according to Beutler et al. (42), and TAC according to Koracevic et al. (40).

### Statistical analysis

Data are presented as mean ± standard error (S.E.). Statistical signifi cance of the data was analyzed using SPSS (version 16). For comparison, one-way ANOVA test and post-comparison was carried out with Duncan’s multiple range test for post hoc analysis. Statistical significance was acceptable to a level of P≤0.05.

## Results

### Results of serum biochemical analysis

**Fig.1:**
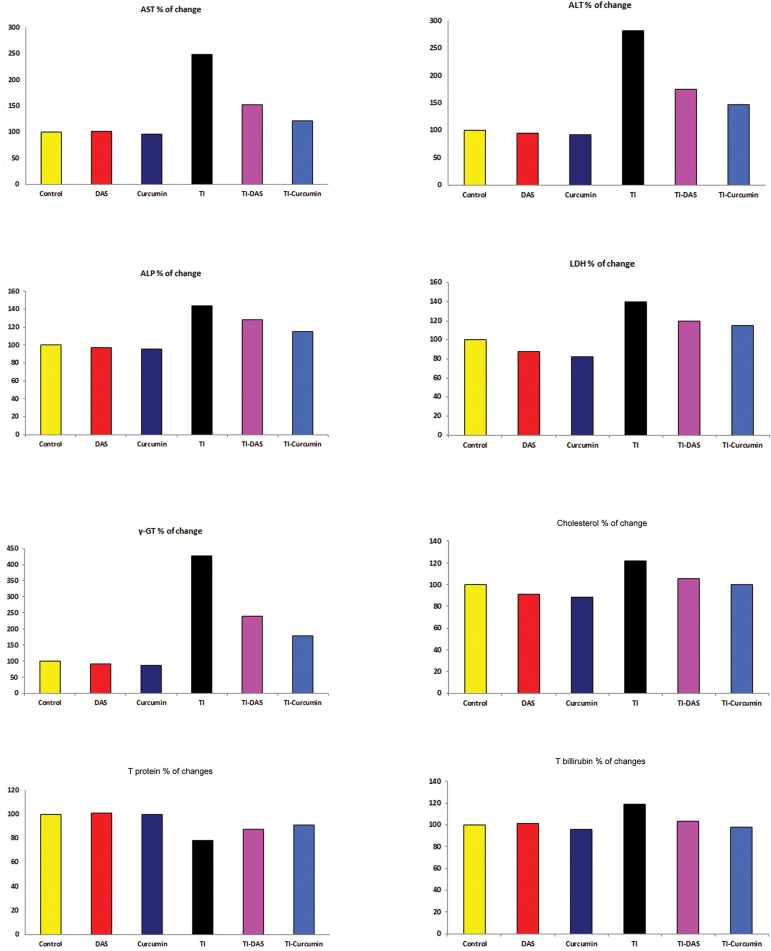
Serum enzyme activities and biochemical parameters in the control and treated groups (percentage of changes from the control group). TI; Thallium acetate, DAS; Diallyl sulfide, AST; Aspartate aminotransferase, ALT; Alanine aminotransferase, ALP; Alkaline phosphatase, LDH; Lactic dehydrogenase, γ-GT; Gamma glutamyltransferase, T protein; Total protein and T bilirubin; Total bilirubin.

The effects of TI intoxication as well as the preventive effects of curcumin and DAS on serum biochemical analysis are shown in table 1 and figure 1. Significant increase (P≤0.05) in serum liver function marker enzymes (AST, ALT, ALP, LDH and γ-GT) was recorded in TI rats compared to the untreated control group. In addition, we observed a significant increase (P≤0.05) in serum cholesterol and total bilirubin, levels. Serum total protein significantly decreased (P≤0.05) in TI rats as compared to the untreated control group.

Pre-treatment with DAS or curcumin one hour prior to TI administration reversed the changes in most studied serum parameters. The results indicated that DAS and curcumin effectively reduced T-induced liver toxicity. In the TI-DAS or TI-curcumin groups, the AST, ALT, ALP, LDH, γ-GT, cholesterol and total bilirubin levels in serum significantly decreased, while serum total protein increased compared with the TI-intoxicated group (P≤0.05).

In the second group (DAS only) serum cholesterol significantly decreased, while in the third group (curcumin only) we found that serum LDH, cholesterol and total bilirubin significantly decreased compared with the untreated non-intoxicated control group (P≤0.05), which indicated the safety of both DAS and curcumin at the selected dose levels in rats.

### Results of serum lipid peroxidation and antioxidant status

**Fig.2:**
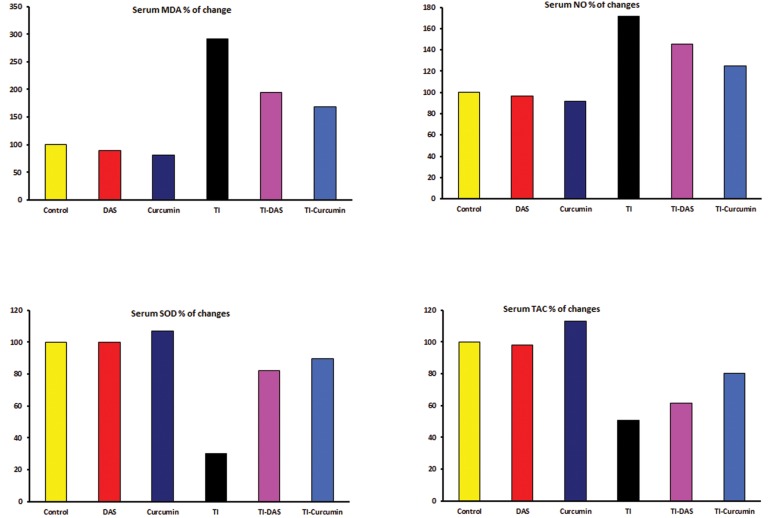
Serum oxidative stress (OS) marker and antioxidant parameters in control and treated groups (percentage of changes from the control group). TI; Thallium acetate, DAS; Diallyl sulfide, MDA; Malondialdehyde, SOD; Superoxide dismutase, NO; Nitric oxide and TAC; Total antioxidant capacity.

The effects of TI intoxication as well as preventive effects of DAS and curcumin on serum lipid peroxidation and antioxidant parameters are shown in table 2 and figure 2. We observed a significant increase (P≤0.05) in serum levels of MDA and NO and a significant decrease (P≤0.05) in serum SOD and TAC levels in TIintoxicated rats compared to the untreated control group.

Pretreatment of intoxicated rats with DAS or curcumin induced a significant decrease (P≤0.05) in serum MDA and NO levels along with a significant increase (P≤0.05) in SOD and TAC levels compared with the TI only group.

### Results of liver lipid peroxidation and antioxidant status

**Fig.3:**
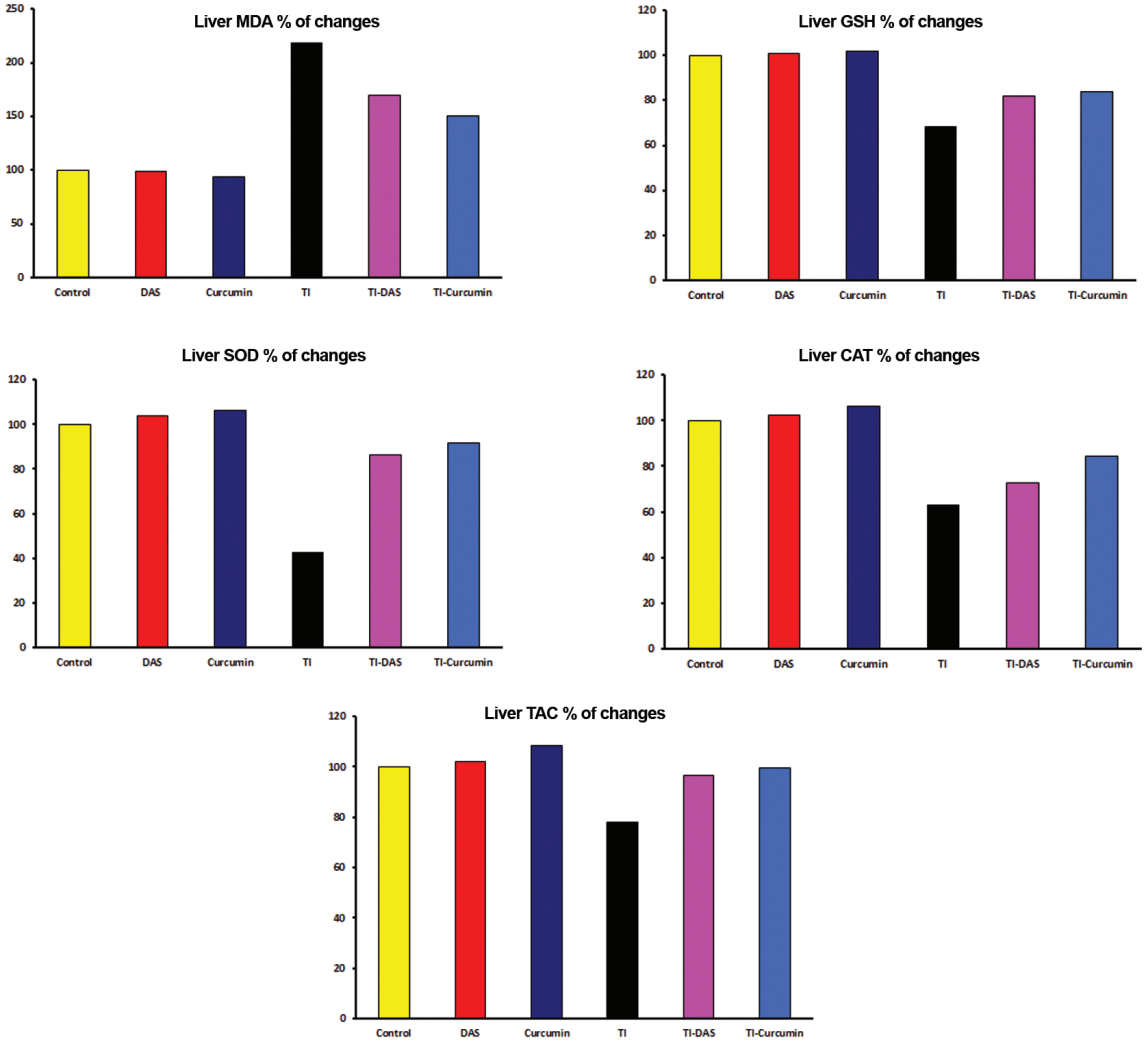
Liver oxidative stress (OS) marker and antioxidant parameters in control and treated groups (percentage of changes from the control group). TI; Thallium acetate, DAS; Diallyl sulfide, MDA; Malondialdehyde, GSH; Reduced glutathione, SOD; Superoxide dismutase, CAT: Catalase and TAC; Total antioxidant capacity.

The effects of TI intoxication as well as the preventive effects of DAS and curcumin on liver tissue homogenate lipid peroxidation and antioxidant parameters are shown in table 3 and figure 3. Significant increase (P≤0.05) in liver MDA content compared with the control group was noticed. On the other hand, liver GSH, SOD, CAT and TAC significantly decreased (P≤0.05).

In the TA-DAS or TA-curcumin group there was a decrease in liver MDA. However GSH, SOD, CAT, and TAC increased compared to the TI-intoxicated group.

**Table 1:** Serum enzyme activities and biochemical parameters in control and treated groups


Parameters	Experimental groups					
	Control	DAS	Curcumin	TI	TI-DAS	TI-curcumin

AST (U/L)	23.8^a^ ± 1.28	24.1^a^ ± 1.17	22.8^a^ ± 1.46	58.9^b^ ± 1.96	36.1^c^ ± 1.76	28.9^a^ ± 1.97
ALT (U/L)	29.9^a^ ± 2.23	28.4^a^ ± 1.426	27.4^a^ ± 1.40	84.4^b^ ± 3.28	52.1^c^ ± 1.23	44.0^c^ ± 1.58
ALP (U/L)	74.3^a^ ± 3.09	72.3^a^ ± 2.95	70.9^a^ ± 2.29	107^b^ ± 2.74	95.5^c^ ± 1.32	85.4^d^ ± 2.17
LDH (U/L)	46.9^a^ ± 1.43	41.0^a^^c^ ± 2.38	38.6^c^ ± 1.08	65.8^b^ ± 1.10	56.0^d^ ± 0.96	53.9^d^ ± 1.60
γ-GT (U/L)	36.3^a^ ± 1.81	33.0^a^ ± 1.25	31.8^a^ ± 0.92	155^b^ ± 5.55	86.5^c^ ± 2.54	64.8^d^ ± 3.29
Cholesterol (mg/dl)	67.1^a^ ± 1.26	61.1^c^ ± 1.04	59.5^c^ ± 1.05	81.6^b^ ± 0.78	70.8^a^ ± 1.47	67.0^a^ ± 2.16
T protein (g/dl)	8.25^a^ ± 0.17	8.33^a^ ± 0.13	8.24^a^ ± 0.17	6.46^b^ ± 0.12	7.23^c^ ± 0.09	7.51^c^ ± 0.14
T bilirubin (mg/dl)	1.48^a^^c^ ± 0.03	1.50^a^^c^ ± 0.02	1.41^c^ ± 0.03	1.76^b^ ± 0.03	1.53^a^ ± 0.02	1.44^a^^c^ ± 0.03


Data are expressed as means ± SE; n=8. TI; Thallium acetate, DAS; Diallyl sulfide, AST; Aspartate aminotransferase, ALT; Alanine aminotransferase, ALP; Alkaline phosphatase, LDH; Lactic dehydrogenase, γ-GT; Gamma glutamyltransferase, T protein; Total protein and T bilirubin; Total bilirubin. Within the same row, different letters indicate statistical significance at P<0.05.

**Table 2:** Serum oxidative stress (OS) marker and antioxidant parameters in control and treated groups


Parameters	Experimental groups					
	Control	DAS	Curcumin	TI	TI-DAS	TI-curcumin

MDA (nmol/L)	2.6^a^ ± 0.22	2.27^a^ ± 0.21	2.05^a^ ± 0.16	7.44^b^ ± 0.36	4.49^c^ ± 0.19	4.28^c^ ± 0.14
NO (μmol/L)	47.9^a^ ± 1.45	46.3^a^ ± 1.49	43.8^a^ ± 1.18	82.1^b^ ± 0.38	69.8^c^ ± 1.50	59.9^d^ ± 1.87
SOD (U/L)	76.1^a^ ± 1.85	76.1^a^ ± 1.72	81.4^e^ ± 1.68	22.9^b^ ± 2.81	62.4^c^ ± 2.24	68.3^d^ ± 1.73
TAC (mmol/L)	1.15^a^ ± 0.04	1.13^a^ ± 0.02	1.30^c^ ± 0.04	0.59^b^ ± 0.03	0.71^d^ ± 0.04	0.92^e^ ± 0.03


Data are expressed as means ± SE; n=8. TI; Thallium acetate, DAS; Diallyl sulfide, MDA; Malondialdehyde, SOD; Superoxide dismutase, NO; Nitric oxide and TAC; Total antioxidant capacity. Within the same row, different letters indicate statistical significance at P<0.05.

**Table 3:** Liver oxidative stress (OS) marker and antioxidant parameters in control and treated groups


Parameters	Experimental groups					
	Control	DAS	Curcumin	TI	TI-DAS	TI-curcumin

MDA (nmol/g)	30.0^a^ ± 1.71	29.6^a^ ± 1.16	28.1^a^ ± 1.08	65.5^b^ ± 1.78	51.0^c^ ± 1.35	45.0^d^ ± 1.45
GSH (mg/g)	55.9^a^ ± 1.49	56.3^a^ ± 0.75	56.9^a^ ± 1.30	38.1^b^ ± 1.19	45.8^c^ ± 1.16	46.8^c^ ± 1.73
SOD (U/g)	759.25^a^ ± 17.2	788.5^a^ ± 15.4	806.63^a^ ± 24.4	321.6^b^ ± 18.9	653.8^c^ ± 9.1	696.1^c^ ± 23.6
CAT (U/g)	0.77^a^ ± 0.03	0.79^a^ ± 0.03	0.82^a^ ± 0.03	0.49^b^ ± 0.01	0.56^c^ ± 0.02	0.65^b^ ± 0.02
TAC (mmol/g)	46.4^a^ ± 1.22	47.4^a^^c^ ± 1.40	50.3^c^ ± 0.65	36.1^b^ ± 0.69	44.9^a^ ± 1.03	46.1^a^ ± 0.81


Data are expressed as means ± SE; n=8. TI; Thallium acetate, DAS; Diallyl sulfide, MDA; Malondialdehyde, GSH; Reduced glutathione, SOD; Superoxide dismutase, CAT; Catalase and TAC; Total antioxidant capacity. Within the same row, different letters indicate statistical significance at P<0.05.

## Discussion

ROS are continuously produced inside the mammalian body as a result of exposure to wide varieties of exogenous chemicals, drugs and xenobiotics in our environment and/or many of endogenous metabolic events that involve the electron transport mechanism and redox enzymes (43-46). In normal situations, an equilibrium exists between the ROS produced and antioxidants that are present as the generated ROS are neutralized by endogenous antioxidants (43, 45, 47). Serious events induced by ROS occur as a consequence of an imbalance between the production and destruction of these species leading to different pathological conditions as well as irregularities in cellular physiology (43, 45, 47, 48). Free radicals have been incriminated in the etiology of many diseases such as cataracts, cancers, stroke, diabetes, coronary heart disease, rheumatoid arthritis, Alzheimer’s disease and the ageing process (49-52).

TI toxicity may be attributed to oxidative tissue damage, which is related to the generation of ROS and OS (4, 25). TI combines with the protein sulfhydryl group and inhibits cellular respiration as well as disruptions to calcium homeostasis. Monovalent TI ions are similar to potassium; therefore, their toxicity may be partially due to interference of membrane channels and transporters associated with potassium (3).

In the present study serum liver function marker enzymes (AST, ALT, γ-GT and ALP) increased in TI intoxicated rats compared to the untreated control group. There was a significant increase in serum total bilirubin, LDH and total cholesterol levels and reduced serum total protein. The biochemical effects of TI were confirmed as shown by an increase in lipid peroxidation; elevation of serum and hepatic MDA and serum NO as well as a reduction in antioxidant parameters; SOD, CAT, GSH in liver and/or serum as well as reduced TAC in both serum and hepatic tissue. These results suggested that the balance between the oxidative and antioxidant systems in rats was disturbed during TI intoxication. Our findings agreed with those by Radic et al. (4) who concluded that TI toxicity was mainly associated with increased ROS formation, tissue damage and dysfunction. In previous studies increased levels of MDA and H_2_O_2_ as well as decreased levels of GSH, GSH-Px and SOD were estimated in animals treated with TI which indicated heavy accumulation of free radicals in the rat liver (3) and brain (25). These results indicated that free radicals might play an important role in the pathogenesis of TI intoxication.

There is an increasing demand for the use of natural compounds that have no adverse effects to alleviate the harmful effects of drugs and xenobiotics (53, 54). In the present study, pre-administration with DAS or curcumin has tended to normalize the increased serum hepatic biomarkers (AST, ALT, ALP, LDH, γ-GT, cholesterol, total bilirubin) and increased the serum total protein level compared in groups 5 and 6, respectively, to the TI-intoxicated non-treated group 4. The enhancement of hepatic biomarkers might be attributed to their antioxidant effect against TI-induced oxidative liver damage. To confirm this hypothesis, lipid peroxidation was evaluated in serum and liver tissue homogenates by estimation of MDA content. We also examined serum and liver SOD and TAC. In addition, serum NO and hepatic tissue CAT and GSH were tested in TI-intoxicated as well as DAS or curcumin-pretreated- TI-intoxicated rats. Curcumin and DAS reduced serum and liver MDA, and serum NO. They also increased serum and liver SOD and TAC as well as liver CAT and GSH. The present results indicated that both DAS and curcumin were excellent natural antioxidant and free radical scavengers; curcumin seemed to be more potent than DAS at the selected doses.

The non-enzymatic antioxidant GSH protects against OS by spontaneous reactions with free radicals. It participates in cell signaling and conjugates xenobiotics, including toxic metals (55). DAS and curcumin, like GSH, may act as freeradical scavengers. TI has a high affinity for –SH groups, thus it may react with GSH and reduce the effective GSH hepatic concentration (24). Interestingly, our results have shown that total GSH levels decreased due to TI toxicity and were signiﬁcantly elevated in group 4 animals compared to the control group. Treatment with DAS and curcumin increased cellular GSH and protected hepatic cells from TI-induced OS.

The pharmacological activity of garlic has been attributed to the presence of organosulfur compounds, mostly related to oil-soluble allyl sulfides such as diallyl trisulfide (DATS), diallyl disulfide (DADS), and DAS. Allyl sulfides showed valuable action in the liver, which could be used for protection of normal liver cells or for improvement of liver damage (56). Numerous researches have approved the antioxidant activity of DAS as it suppressed cytotoxicity and oxidative tissue damage by increasing antioxidant enzyme activities and decreasing lipid peroxidation. Therefore, it can be used as a dietary preventive agent (11, 12, 17, 57).

Curcumin, a phytochemical found in the spice turmeric has been used for centuries without any known adverse effects. It has some useful effects against a variety of chronic diseases. Many of these therapeutic actions can be due to its powerful antioxidant and anti-inflammatory activities, and therefore may be potentially used to combat OS (58). Curcumin has the ability to suppress lipid peroxidation and ameliorate OS, therefore it increases the xenobiotic detoxifying ability of the tissues (21-23).

There was an association between TI toxicity and free radical-mediated OS demonstrated by increased levels of MDA and NO as well as decreased levels of antioxidant parameters in the rats' sera and livers. Pre-administration of DAS and curcumin played an important role in the prevention of TI-induced OS and boosted the cellular antioxidant defense system. These effects were probably through their antioxidant and free radical scavenging capacities. Finally, DAS and curcumin could be suggested for human and animals at risk of TI exposure. They might be helpful for those who already suffer from TI intoxication.

## References

[1] Hwang MH, Damte D, Cho MH, Kim YH, Park SC (2010). Optimization of culture media of pathogenic Mycoplasma hyopneumoniae by a response surface methodology. J Vet Sci.

[2] Puschner B, Basso MM, Graham TW (2012). Thallium toxicosis in a dog consequent to ingestion of Mycoplasma agar plates. J Vet Diagn Invest.

[3] Kilic GA, Kutlu M (2010). Effects of exogenous metallothionein against thallium-induced oxidative stress in rat liver. Food Chem Toxicol.

[4] Radic S, Cvjetko P, Glavas K, Roje V, Pevalek-Kozlina B, Pavlica M (2009). Oxidative stress and DNA damage in broad bean (Vicia faba L.) seedlings induced by thallium. Environ Toxicol Chem.

[5] Mayuren C, Reddy VV, Priya SV, Devi VA (2010). Protective effect of Livactine against CCl(4) and paracetamol induced hepatotoxicity in adult Wistar rats. N Am J Med Sci.

[6] Abdel-Daim MM, Ghazy EW, Fayez M (2015). Synergistic protective role of mirazid (Commiphora molmol) and ascorbic acid against tilmicosin-induced cardiotoxicity in mice. Can J Physiol Pharmacol.

[7] Abdel-Daim MM, Abd Eldaim MA, Mahmoud MM (2014). Trigonella foenum-graecum protection against deltamethrin-induced toxic effects on haematological, biochemical, and oxidative stress parameters in rats. Can J Physiol Pharmacol.

[8] Abdel-Daim MM (2014). Pharmacodynamic interaction of Spirulina platensis with erythromycin in Egyptian Baladi bucks (Capra hircus). Small Ruminant Res.

[9] Heo BG, Park YS, Chon SU, Lee SY, Cho JY, Gorinstein S (2007). Antioxidant activity and cytotoxicity of methanol extracts from aerial parts of Korean salad plants. Biofactors.

[10] Chen GW, Chung JG, Ho HC, Lin JG (1999). Effects of the garlic compounds diallyl sulphide and diallyl disulphide on arylamine N-acetyltransferase activity in Klebsiella pneumoniae. J Appl Toxicol.

[11] Grudzinski IP, Frankiewicz-Jozko A, Bany J (2001). Diallyl sulfide--a flavour component from garlic (Allium sativum) attenuates lipid peroxidation in mice infected with Trichinella spiralis. Phytomedicine.

[12] Yin MC, Hwang SW, Chan KC (2002). Nonenzymatic antioxidant activity of four organosulfur compounds derived from garlic. J Agric Food Chem.

[13] Hassan HA, Hafez HS, Zeghebar FE (2010). Garlic oil as a modulating agent for oxidative stress and neurotoxicity induced by sodium nitrite in male albino rats. Food Chem Toxicol.

[14] Pedraza-Chaverri J, Gonzalez-Orozco AE, Maldonado PD, Barrera D, Medina-Campos ON, Hernandez-Pando R (2003). Diallyl disulfide ameliorates gentamicin-induced oxidative stress and nephropathy in rats. Eur J Pharmacol.

[15] Pedraza-Chaverri J, Maldonado PD, Barrera D, Ceron A, Medina-Campos ON, Hernandez-Pando R (2003). Protective effect of diallyl sulfide on oxidative stress and nephrotoxicity induced by gentamicin in rats. Mol Cell Biochem.

[16] Iranloye BO, Oludare GO (2011). Garlic and vitamin E provides antioxidant defence in tissues of female rats treated with nicotine. Niger J Physiol Sci.

[17] Ho CY, Cheng YT, Chau CF, Yen GC (2012). Effect of diallyl sulfide on in vitro and in vivo Nrf2-mediated pulmonic antioxidant enzyme expression via activation ERK/p38 signaling pathway. J Agric Food Chem.

[18] Araujo CC, Leon LL (2001). Biological activities of Curcuma longa L. Mem Inst Oswaldo Cruz.

[19] Chainani-Wu N (2003). Safety and anti-inflammatory activity of curcumin: a component of turmeric (Curcuma longa). J Altern Complement Med.

[20] Surh YJ, Chun KS, Cha HH, Han SS, Keum YS, Park KK (2001). Molecular mechanisms underlying chemopreventive activities of anti-inflammatory phytochemicals: down-regulation of COX-2 and iNOS through suppression of NF-kappa B activation. Mutat Res.

[21] Okada K, Wangpoengtrakul C, Tanaka T, Toyokuni S, Uchida K, Osawa T (2001). Curcumin and especially tetrahydrocurcumin ameliorate oxidative stress-induced renal injury in mice. J Nutr.

[22] Rukkumani R, Aruna K, Varma PS, Menon VP (2004). Curcumin influences hepatic expression patterns of matrix metalloproteinases in liver toxicity. Ital J Biochem.

[23] Iqbal M, Sharma SD, Okazaki Y, Fujisawa M, Okada S (2003). Dietary supplementation of curcumin enhances antioxidant and phase II metabolizing enzymes in ddY male mice: possible role in protection against chemical carcinogenesis and toxicity. Pharmacol Toxicol.

[24] Villaverde MS, Hanzel CE, Verstraeten SV (2004). In vitro interactions of thallium with components of the glutathionedependent antioxidant defence system. Free Radic Res.

[25] Galvan-Arzate S, Pedraza-Chaverri J, Medina-Campos ON, Maldonado PD, Vazquez-Roman B, Rios C (2005). Delayed effects of thallium in the rat brain: regional changes in lipid peroxidation and behavioral markers, but moderate alterations in antioxidants, after a single administration. Food Chem Toxicol.

[26] Hanzel CE, Villaverde MS, Verstraeten SV (2005). Glutathione metabolism is impaired in vitro by thallium(III) hydroxide. Toxicology.

[27] Rios C, Monroy-Noyola A (1992). D-penicillamine and prussian blue as antidotes against thallium intoxication in rats. Toxicology.

[28] Farombi EO, Shrotriya S, Na HK, Kim SH, Surh YJ (2008). Curcumin attenuates dimethylnitrosamine-induced liver injury in rats through Nrf2-mediated induction of heme oxygenase-1. Food Chem Toxicol.

[29] Reitman S, Frankel S (1957). A colorimetric method for the determination of serum glutamic oxalacetic and glutamic pyruvic transaminases. Am J Clin Pathol.

[30] Tietz NW, Burtis CA, Duncan P, Ervin K, Petitclerc CJ, Rinker AD (1983). A reference method for measurement of alkaline phosphatase activity in human serum. Clin Chem.

[31] Lowry OH, Rosebrough NJ, Farr AL, Randall RJ (1951). Protein measurement with the Folin phenol reagent. J Biol Chem.

[32] Babson SR, Babson AL (1973). An improved amylase assay using dyed amylopectin. Clin Chim Acta.

[33] Szasz G (1969). A kinetic photometric method for serum gammaglutamyl transpeptidase. Clin Chem.

[34] Richmond W (1973). Preparation and properties of a cholesterol oxidase from Nocardia sp.and its application to the enzymatic assay of total cholesterol in serum. Clin Chem.

[35] Allain CC, Poon LS, Chan CS, Richmond W, Fu PC (1974). Enzymatic determination of total serum cholesterol. Clin Chem.

[36] Schmidt M, Eisenburg J (1975). Serum bilirubin determination in newborn infants.A new micromethod for the determination of serum of plasma bilirubin in newborn infants. Fortschr Med.

[37] Mihara M, Uchiyama M (1978). Determination of malonaldehyde precursor in tissues by thiobarbituric acid test. Anal Biochem.

[38] Green LC, Wagner DA, Glogowski J, Skipper PL, Wishnok JS, Tannenbaum SR (1982). Analysis of nitrate, nitrite, and [15N] nitrate in biological fluids. Anal Biochem.

[39] Nishikimi M, Appaji N, Yagi K (1972). The occurrence of superoxide anion in the reaction of reduced phenazine methosulfate and molecular oxygen. Biochem Biophys Res Commun.

[40] Koracevic D, Koracevic G, Djordjevic V, Andrejevic S, Cosic V (2001). Method for the measurement of antioxidant activity in human fluids. J Clin Pathol.

[41] Aebi H (1984). Catalase in vitro. Methods Enzymol.

[42] Beutler E, Duron O, Kelly BM (1963). Improved method for the determination of blood glutathione. J Lab Clin Med.

[43] Madkour FF, Abdel-Daim MM (2013). Hepatoprotective and antioxidant activity of Dunaliella salina in paracetamolinduced acute toxicity in rats. Indian J Pharm Sci.

[44] Azab S, Abdel-Daim M, Eldahshan O (2013). Phytochemical, cytotoxic, hepatoprotective and antioxidant properties of Delonix regia leaves extract. Med Chem Res.

[45] Abdel-Daim M, Halawa S (2014). Synergistic hepatocardioprotective and antioxidant effects of myrrh and ascorbic acid against diazinon-induced toxicity in rabbits. Int Res J Humanit Eng Pharm Sci.

[46] Al-Sayed E, Martiskainen O, Seif el-Din SH, Sabra A-NA, Hammam OA, El-Lakkany NM (2014). Hepatoprotective and antioxidant effect of bauhinia hookeri extract against carbon tetrachloride-induced hepatotoxicity in mice and characterization of its bioactive compounds by HPLCPDA- ESI-MS/MS. Biomed Res Int.

[47] Sun Y (1990). Free radicals, antioxidant enzymes, and carcinogenesis. Free Radic Biol Med.

[48] Eldahshan OA, Abdel-Daim MM (2014). Phytochemical study, cytotoxic, analgesic, antipyretic and anti-inflammatory activities of Strychnos nux-vomica. Cytotechnology.

[49] Abdel-Daim M, Funasaka Y, Kamo T, Ooe M, Matsunaka H, Yanagita E (2010). Preventive effect of chemical peeling on ultraviolet induced skin tumor formation. J Dermatol Sci.

[50] Funasaka Y, Abdel-Daim M, Kawana S, Nishigori C (2012). Effect of chemical peeling on the skin in relation to UV irradiation. Exp Dermatol.

[51] Abdel-Daim M, Funasaka Y, Kamo T, Ooe M, Matsunaka H, Yanagita E (2010). Effect of chemical peeling on photocarcinogenesis. J Dermatol.

[52] Willcox JK, Ash SL, Catignani GL (2004). Antioxidants and prevention of chronic disease. Crit Rev Food Sci Nutr.

[53] Al-Sayed E, Abdel-Daim MM (2014). Protective role of cupressuflavone from Cupressus macrocarpa against carbon tetrachloride-induced hepato- and nephrotoxicity in mice. Planta Med.

[54] Abdou RH, Abdel-Daim MM (2014). Alpha-lipoic acid improves acute deltamethrin-induced toxicity in rats. Can J Physiol Pharmacol.

[55] Verstraeten SV (2006). Relationship between thallium(I)-mediated plasma membrane fluidification and cell oxidants production in Jurkat T cells. Toxicology.

[56] Iciek MB, Kowalczyk-Pachel D, Kwiecien I, Dudek MB (2012). Effects of different garlic-derived allyl sulfides on peroxidative processes and anaerobic sulfur metabolism in mouse liver. Phytother Res.

[57] Chen L, Hong JY, So E, Hussin AH, Cheng WF, Yang CS (1999). Decrease of hepatic catalase level by treatment with diallyl sulfide and garlic homogenates in rats and mice. J Biochem Mol Toxicol.

[58] Meng B, Li J, Cao H (2013). Antioxidant and antiinflammatory activities of curcumin on diabetes mellitus and its complications. Curr Pharm Des.

